# Challenges of Internet Recruitment: A Case Study with Disappointing Results

**DOI:** 10.2196/jmir.7.1.e6

**Published:** 2005-03-19

**Authors:** Malcolm Koo, Harvey Skinner

**Affiliations:** ^1^University of TorontoDepartment of Public Health SciencesFaculty of MedicineToronto, ONCanada

**Keywords:** Internet, data collection, World Wide Web, research subjects

## Abstract

**Background:**

The Internet provides tremendous opportunities for innovative research, but few publications on the use of the Internet for recruiting study participants exist. This paper summarizes our experiences from 2 studies in which we attempted to recruit teenagers on the Internet for a questionnaire study to evaluate a smoking-cessation website.

**Objective:**

To evaluate strategies of recruiting teenagers for the evaluation of a smoking-cessation website through the Internet.

**Methods:**

In Study 1 (Defined Community Recruitment), we sent invitation emails to registered members of a youth health website, CyberIsle. A total of 3801 email addresses were randomly divided into 2 groups. In the first group, emails indicated that the first 30 respondents would receive a Can $20 electronic gift certificate for use at an online bookstore if they would go to the Smoking Zine website and respond to a short survey. For the second group, the email also indicated that respondents would receive an additional Can $10 gift certificate if they referred their friends to the study. Reminder emails were sent 10 days after the sending of the initial invitation email. In Study 2 (Open Recruitment), we posted invitation messages on Web discussion boards, Usenet forums, and one specialized recruitment website, and attempted a snowball recruiting strategy. When potential participants arrived at the study site, they were automatically randomized into either the higher incentives group (Can $15 electronic gift certificate) or lower incentive group (Can $5 gift certificate).

**Results:**

In Study 1 (defined community recruitment), 2109 emails were successfully delivered. Only 5 subjects (0.24%), including 1 referred by a friend, passed the recruitment process and completed the questionnaire; a further 6 individuals visited the information page of the study but did not complete the study. In Study 2 (open recruitment), the number of users seeing the advertisement is unknown. A total of 35 users arrived at the website, of whom 14 participants were recruited (8 from the Can $15 gift certificate group and 6 from the Can $5 gift certificate group). Another 5 were recruited from the general Internet community (3 from discussion boards and 2 from the Research Volunteers website). The remaining 9 participants were recruited through friend referrals with the snowball strategy.

**Conclusions:**

Overall, the recruitment rate was disappointingly low. In our case, recruitment using Internet technologies including email, electronic discussion boards, Usenet forums, and websites did not prove to be an effective approach for soliciting young subjects to participate in our research. Possible reasons are discussed, including the participants' perspective. A major challenge is to differentiate trustable and legitimate messages from spam and fraudulent misinformation on the Internet. From the researchers' perspective, approaches are needed to engage larger samples, to verify participants' attributes, and to evaluate and adjust for potential biases associated with Internet recruitment.

## Introduction

The advent of the Internet has radically changed communication and information dissemination patterns among individuals and in society at large. Internet services such as websites, email, newsgroups, and blogs are providing new and powerful ways of disseminating and collecting information. Researchers have long been aware of the potential of the Internet [1,2]. The Internet has been considered a promising media for teaching and learning [3], research communications [1], and dissemination of medical information [4]. More recently, advancement in Web technology and its widespread adoption have further fostered the innovative use of the Internet in the areas of data collection [5-7] and online intervention programs and experimental research [8,9].

However, few published reports on the experiences of using the Internet for recruiting study participants are available [10-12]. Some authors have expressed concerns on the unrepresentativeness of Internet samples. Etter and Perneger [13] compared study participants who were recruited through a French-language smoking-cessation website with those recruited by mail. They found that smokers recruited through the Internet were younger, more educated, and more motivated to quit smoking; they also smoked more cigarettes per day than smokers in the other group. Despite the difference in smokers' characteristics, the authors concluded that Internet recruitment is a potentially useful method for analytical studies, which focus on associations between variables, but not for descriptive studies. Another study [14] evaluated whether the Internet could help to shorten the patient recruitment process in clinical trials. The authors concluded that the Internet is unlikely to become the core recruitment medium in the near future, but may be used as a part of an integrated approach to recruitment, mainly to inform potential participants of recruitment opportunities. The lack of representativeness of self-referred volunteers (they tend to be better educated, younger, and non-immigrants) threatens external validity--a major concern for an Internet-based recruitment approach for clinical trials.

Since young people are generally the early adopters of new technologies, the Internet holds great promise as an innovative medium for health research with this population [15].

In this paper, we first present results from two studies on the effectiveness of using the Internet to recruit young participants and then discuss some of the main challenges for Internet recruitment. The aim is to report our experiences on using the Internet for recruiting participants in studies. To present results from the Web-based studies themselves is not within the scope of this paper.

## Methods

### Study 1: Defined Community Recruitment

We sent invitation email messages to a subset of registered members of the CyberIsle youth website [16,17] during March 2003. The CyberIsle website [18] is a comprehensive Web-based health resource developed by the TeenNet Research Program [19], a youth health promotion initiative based at the University of Toronto. Subjects were selected from the registered member database if they were between 12 and 24 years old at the time of our study, resided in Canada, had provided their email addresses, and agreed to be researched for their activities on the CyberIsle website. Smoking status was not a selection criterion in the study.

**Figure 1:**
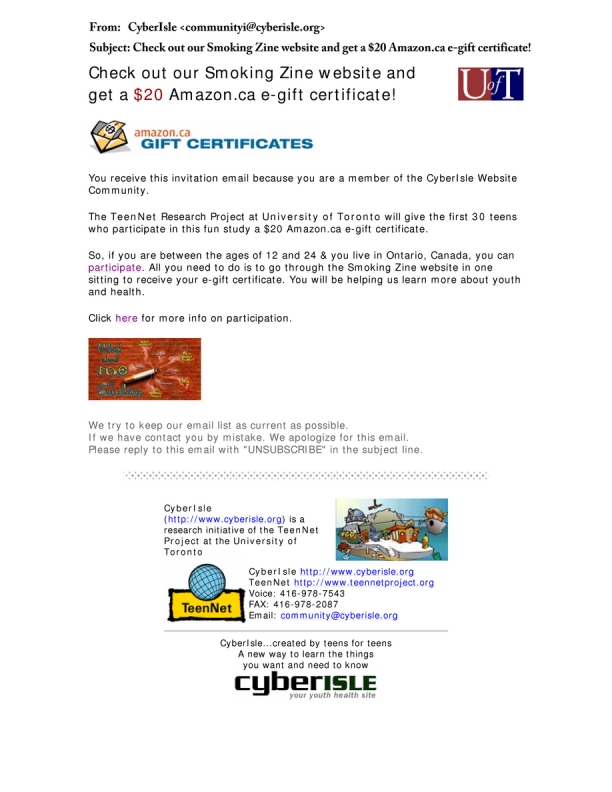
Invitation email written in hypertext markup language used in Study 1

The resulting 3801 email addresses were randomly divided into 2 groups. In the first group, emails indicated that the first 30 respondents would receive a Can $20 electronic gift certificate for use at an online bookstore if they went to the Smoking Zine website and responded to a short online survey (Figure 1). We decided to offer incentives only to the first 30 respondents to minimize the reaction time of the participants to the invitation. The Smoking Zine [20] is a Web-based smoking prevention and cessation intervention for youth that is embedded in the CyberIsle. In the second group, the invitation email also indicated that respondents would receive an additional Can $10 gift certificate if they referred at least one friend  to the study. Thus, respondents could receive as a maximum gift certificates in the amount of Can $30 if they were able to refer a friend (or multiple friends) to the study.

In both groups, the invitation email was written in hypertext markup language (HTML) and contained images including a prominent banner depicting a Can $20 gift certificate, a logo of the University of Toronto, and screen images of the front page of the Smoking Zine and the CyberIsle websites (Figure 1). In addition, hyperlinks leading to the study website, contact information including telephone number and email address, and instructions for opting out of further email contact were provided. Bounced emails as a result of invalid email addresses were removed from the study email database.

**Figure 2:**
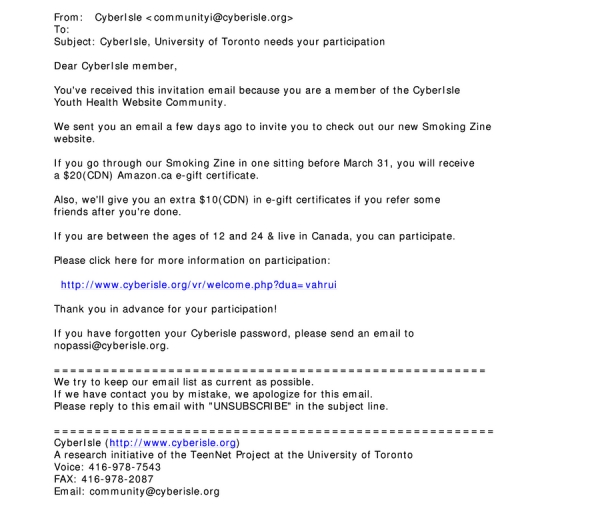
Reminder email written in plain text used in Study 1

Reminder emails were sent 10 days after the initial invitation email. The reminder email messages were fully text-based (not HTML-based) and no graphical images were used (Figure 2). The study was terminated 10 days after the reminder email.

When potential participants clicked the hyperlink on the invitation email, they connected to a Web page containing information about the study and a consent form. To proceed with the study, participants were required to click a button to indicate their consent to the online study. After going through the stage-based Smoking Zine website, participants were automatically presented with a short online 18-item questionnaire with 17 closed-ended multiple-choice questions on their Internet behavior and experience with the Smoking Zine. One open-ended question was placed at the end of the survey for participants to provide general comments. No sociodemographic data were collected from the participants. Once participants completed the questionnaire, they were sent an email indicating that they would receive the electronic gift certificates by email. During the study, participants who tried to exit the website without completing all 5 stages of the Smoking Zine would automatically be presented with a short 5-item questionnaire.

### Study 2: Open Recruitment

Subject recruitment from the general Internet population was evaluated during March 2004. Invitations to participate were posted on Web discussion boards that were relevant to youth and smoking. The posting indicated that individuals must be between 15 to 24 years of age at the time of our study and residing in Canada to be eligible for the electronic gift certificate for participation. We selected 2 Canadian websites designed for youth (TakingITGlobal and Spank!) that had discussion boards with topics related to health and smoking [21,22]. In addition, Usenet forums were identified through Google Groups [23] where users were likely to have some ties with the University of Toronto community. The intention was to improve the credibility of our posting by choosing an audience that was local to our research project. The forums included were ut.general and ut.chinese. Also, we posted in a general smoking-related Usenet forum (alt.quit.smoking.support) that is not geographically restricted to Canada, as well as at the discussion board from the website of a local University of Toronto student group [24]. Finally, our posting was submitted to a new website, Research Volunteers [25], designed specifically for recruiting study participants through the Web. At the time of our study, the Research Volunteers website had been open to the public for one month and there were 9 studies in the database (8 from Ontario and 1 from British Columbia).

The message posted on the boards and forums was text-based and contained a link to our study website. There is no way of knowing how many people saw the advertisement. On all of the boards and forums except one, the message was posted for up to 24 days. However, on one board (Spank!) [22] our message was removed by the board administrator within a few minutes of being posted because it was perceived as spam [26] and had violated their discussion board rules.

When potential participants entered the study site, they were automatically randomized into either the higher incentives group (Can $15 electronic gift certificate for completing the Smoking Zine and a Web-based survey) or the lower incentives group (Can $5 gift certificate for completing the Smoking Zine). In both groups, participants had the opportunity to get additional $10 gift certificates for providing email addresses for up to 5 friends in respective fields presented after they filled in the survey.

Snowball sampling through referral by friends was also evaluated in this study. We asked 1 young subject who had been involved with other TeenNet evaluations as the initial recruiter to send personal emails to 8 of her friends with a message indicating that an invitation email from our study would be sent to them soon. To remind the recipients that our email was the one mentioned by their friend, the initial referrer's email address was indicated in the message. We hoped that the 8 participants would each suggest up to 5 friends after filling in the survey, that these 5 would suggest 5 other friends, and so on.

The research protocol was approved by the University of Toronto's Human Subjects Review Committee. In both studies, participants were only required to provide their email addresses in order to receive the electronic gift certificate. In order to ensure anonymity of the participants' identity, no other contact information such as names, mailing addresses, or phone numbers was collected.

## Results

### Study 1: Defined Community Recruitment

In the first study, 3801 recruitment emails were sent to members of the health website, CyberIsle. Of those, 1692 emails were undeliverable and the maximum number of youth who had possibly received our email was 2109. A total of 5 subjects (0.24%) satisfied recruitment criteria and completed the questionnaire; a further 6 individuals visited the information page of the study but did not proceed to the recruitment stage.

Initially, no response was received in response to the first email. After the reminder email was sent out, 4 participants (0.2%) completed the study (1 was from the first group and 3 were from the second group, in which participants received an additional Can $10 gift certificate for referring a friend). In the second group 1 participant referred 5 friends to the study, of whom 1 completed the study.

### Study 2: Open Recruitment

**Figure 3:**
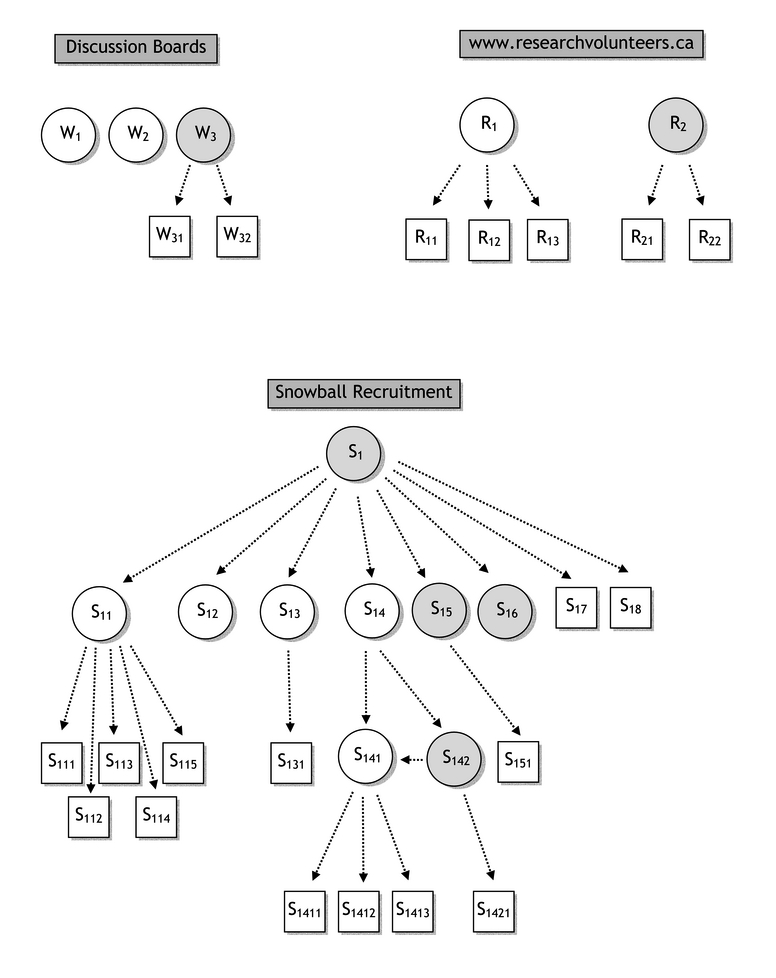
The referral patterns of the three recruitment sources in Study 2

In the second study, several routes of recruitment were attempted including Web discussion boards, Usenet forums, and a specialized recruitment website. A total of 14 participants were recruited of whom 5 were from the general Internet community (3 from discussion boards [subjects labeled with W] and 2 from the Research Volunteers [R] website). The remaining 9 participants were recruited through friend referrals using the snowball strategy [S]. Figure 3 shows the referral patterns with the levels of incentives. Eight participants received Can $15 gift certificates (unshaded circles), and 6 participants received Can $5 gift certificates (shaded circles). Those who were referred by their friends but did not participate in the study are indicated with unshaded squares.

Participants who received Can $15 gift certificates are indicated with unshaded circles; those who received Can $5 gift certificates are indicated with shaded circle; and those who were referred by their friends but did not participate in the study are indicated with unshaded squares.

Despite the potential of receiving an additional Can $10 gift certificate, 4 participants (W_1_, W_2_, S_12_, S_16_) did not provide any email addresses (see Figure 3). Although 3 of the participants from the discussion board and Research Volunteers website (W_3_, R_1_, R_2_) provided referrals (W_31_, W_32_, R_11_, R_12_, R_13_, R_21_, R_22_), none of these 7 individuals responded to our invitation message to participate.

In the snowball recruitment (see Figure 2), 6 out of 8 (75%) individuals from the first level of referral responded and joined the study. Of these, 4 out of 6 (67%) provided email addresses for a total of 9 friends. At the second level of referral, only 2 out of these 9 (22%) participated and provided email addresses for a total of 5 friends, of whom one was a participant who had already enrolled (S_141_). At the third level, 0 out of 4 were recruited and none provided further email addresses. The good response at the first level of referrals was the result of the initial referrer (S_1_) sending personal emails to each of the 8 individuals to indicate the coming of our study email. If the email from the initial referrer had not been received, 2 participants would not have received the initiation email because their Web-based email accounts, Hotmail [27], were set so that unless the sender's email addresses already existed in the participant's personal address list, the email would be sent to the junk mail box.

Over the entire open recruitment study, 35 visitors arrived at the study website. Since the total number of visitors was 35 and the number of actual participants in our study was 14, the overall participation rate was 14 out of 35 or 40%. However, it must be noted that 10 participants arrived at the study page through referrals. These were more likely to participate than those who visited the site because of seeing our posting on discussion boards. Therefore, assuming all participants that came from referrals joined the study, a more conservative estimate of the actual participation for those who had reached the study website should be 4 out of 25 (16%).

Regarding the characteristics of the 14 participants, 12 (86%) used email everyday, 11 (79%) used the Web everyday, and 9 (64%) used instant messaging everyday. As expected, youth often actively maintained more than one email account [28]. The majority of participants (8/14, or 57%) used 3 or more email accounts.

## Discussion

In our studies we experienced very low participation rates, despite the provision of monetary incentives. Since potential participants in the first study were members of our research website, CyberIsle, we did not expect such a low participation rate (0.2%) to our email invitation. This figure is close to the lower bound of response rates from email marketing of 0.1% [29] rather than the average rate of 1%. Several possible explanations for the low participation are discussed below followed by a description of some of the challenges of Internet recruitment.

### Authenticity and Legitimacy of Information on the Internet

With the large number of websites youth encounter, it is plausible that the email recipients did not remember their previous involvement with CyberIsle*.* They may have considered the recruitment message as unsolicited commercial mass email (spam). Our initial recruitment email, which had a response rate of 0%, was formatted in HTML with colors and embedded images (Figure 1). The graphical layout along with several hyperlinks might have been mistaken for spam by the recipients or by the built-in spam filter in email programs resulting in automatic deletion from the incoming mailbox. However, the response was still low even when the reminder email was formatted as plain text (Figure 2). The recipients may not have received our second email because of spam filters or because they did not regularly check the email account of the address they provided during the CyberIsle registration (youth often set up separate email accounts used specifically for registration purposes).

Given the low response rate from the first study, where the potential participants were members of our health website, it is not surprising to see a similar low response rate when we extended the recruitment to the general Internet community where there had been no previous connection with our research project.

The level of spam and deceptive email on the Internet has exploded exponentially in the past few years [30]. The spam to non-spam ratio as of March 2004 was estimated to be 63%. About 12% of spam was estimated to be scams or fraud and many were infected with viruses or worms [31] that pose a serious threat to online privacy. Since online privacy is one of the major concerns for youth online, it is not surprising that postings or email messages that bear even slight resemblance to spam are ignored.

The context of a message may influence the decision of potential participants to join a study. We expected that postings on University of Toronto-related Usenet forums and discussion boards would enhance the credibility and relevancy of our study. However, only 3 participants were from the University of Toronto discussion boards. It is possible that more individuals would have participated in the study if we had kept the postings online for a longer period of time. However, older messages on discussion boards are rarely browsed once they are not shown on the first page (pushed to later pages by newer postings).

### Incentives

The incentives level might not have been sufficient or gift certificates for an online bookstore may not have been attractive enough for our young potential participants. One of the limitations of electronic gift certificates is that the price of purchase must be lower than the value of the gift certificate. Otherwise, one would need to have access to a credit card in order to purchase online, which is an issue for trials with teenagers. After allowing for taxes and shipping charges, a Can $20-dollar certificate is worth only about Can $14 thereby limiting what can be bought. Despite this limitation with electronic gift certificates, it was chosen as the incentive in the study because of the anonymity it provided. Only a valid email address is required to deliver a certificate to a participant, as opposed to requiring the postal address if other coupons usable in stores are used as incentives. Until electronic cash payments such as PayPal become widely accepted, there are limited options for compensating respondents for their participation in an anonymous way.

### Snowball Sampling and Personalization

The explosion of spam on the Internet may explain why our snowball recruitment through email referrals was ineffective. Despite the potential of receiving an additional Can $10 of gift certificate, 4 of the 14 participants in the second study did not provide their friends' email addresses. This is not surprising since they might have wished to preserve their friends' privacy.

Recruitment emails sent to the referrals in both studies were addressed from our study email account. In the body of the email, we indicated that how and from whom (email address of the referrer provided) we had obtained the referrals' email addresses. In the same email, we also sent a copy to the referrer as a way of indicating the legitimacy of the email. Additional personalization to the email was not introduced since we had only the email addresses of the referrals.

A recent study on online shoppers found that compared to basic site improvements such as ease of navigation, the effect of personalization provided little incentive for users to buy from an e-commerce website [32]. This is in contrast to the general recommendation given to improve response rates in mail surveys [33]. Again, the weak effect of personalization in email could be the result of widespread personalization in most electronic marketing materials encountered on a daily basis. Better response might be achieved if recruitment emails were sent directly under the referrers' email addresses rather than from the study email address. Instead of sending the referral's email to the study coordinator, the study website could be programmed so that the referrers could send invitation emails using their own email addresses directly to their friends. Spammers have exploited various deceptive techniques such as employing fake sender email addresses from legitimate domains, embedding real logos from legitimate websites onto messages, and using misleading or enticing (such as money or free prizes) subject lines. Therefore it is almost impossible to create a recruitment email message or a Web posting that can easily be distinguished from spam by a casual Internet user. For email to be a viable recruitment medium, more research is needed to explore the factors contributing to a trustable message.

### Challenges and Practical Advice

#### Verification of Participants' Attributes

Because of the anonymous nature of our study design, it was impossible to verify the age of the participants. The eligible age range for our second study was 15 to 24 years. There is no simple online solution for verifying an Internet user's true age. A 2001 study on youth Internet behavior found that 15% of online teens and 25% of older boys when online have lied about their age to gain access to websites which often are pornographic in nature [28]. On the other hand, in studies where adult participants are required, it is possible to use commercial online age verification services using credit card information as the verifying identifier. However, privacy issues will become a major concern as individually identifiable information is collected by age verification companies.

The information page of our study specified that enrolment was limited to individuals currently living in Canada. There is no simple way to check or enforce the geographical location of a participant, although it is possible, with various free reverse lookup Internet websites, to identify the country of a participant's computer using the Internet Protocol (IP) address. However, it is both difficult and costly to implement this as a real-time check feature on the site. The solution we adopted in this study was to target our postings only to Canadian discussion boards and Usenet forums.

#### Preventing Multiple Participation

Preventing multiple entries from the same participant is another challenge for Internet recruitment, particularly in studies with monetary incentives [5,34]. Since it is simple for anyone to apply for new email accounts from free email service providers such as the Hotmail and the Yahoo Mail, the same individual can create multiple identities and participate in a study more than once. This issue is particularly difficult in studies using discussion boards or Usenet forums where unique login information (username/password) or URL cannot be assigned to each participant. The use of cookies is only effective to detect multiple participations if the participants access the study website twice from the same computer. This detection method can easily be circumvented by using a different computer or by deleting the cookies from the computer.

One step which can identify multiple participation is the examination of the survey results submitted from same IP addresses for the presence of other indications for multiple participation, such as the lack of internal consistency between items in the survey, and unrealistically short response time to survey questions [5].

Simply deleting all entries with duplicate IP addresses is not recommended, because the recent popularity of proxy servers or network address translation (NAT) servers, have made it not uncommon for one public IP address to be shared across many computers within a private local area network [35]. In addition, for computers connecting to the Internet through dynamic IP addresses (dial-up or broadband), new IP address can be obtained simply by logging in again. Thus, duplicate IP addresses do not necessarily indicate multiple entries from the same person and to delete all such entries would eliminate legitimate data.

Reips [9] estimates that repeat participations were below 3% in most studies and should not be a threat to the data quality of Internet-based research.

### Coverage

Participation in Internet recruitment may be increased by broadening the dissemination of the recruitment information. For example, one can post the study invitation on those discussion boards or Usenet forums that have higher posting traffic, such as those related to computers. However, it is bad “netiquette” to cross-post in forums with out-of-context messages, such as study recruitment of a health behavior study in a computer-related forum. Such messages will either be ignored or removed. In some cases, the sender will be “flamed” (responded to by overly harsh and often hostile terms). Another possibility is to purchase advertisement space such as in the form of page banner on websites that are popular among target users.

### Conclusion

This study is one of the first attempts to investigate the feasibility of Internet recruitment in the “age of spam.” In our specific case our recruitment strategies were not efficient. However, we caution against generalizing our negative results. Internet recruitment may prove viable if studies are conducted on a larger scale, if the right newsgroups are targeted, the right incentives chosen, and the right wording is used. Recruitment announcements in the form of Web page banners can potentially be viewed by tens of thousands, if not more, of online users on high traffic Web portals.

From the researchers' perspective, the validity of study results can be compromised by limitations in verifying participants' attributes such as age. For motivated participants, it is not clear how to differentiate trustable and legitimate messages on the Internet. Researchers using Internet recruitment in their studies should focus on ways to improve the perceived legitimacy of the invitation message. For example, participants should be able to easily identify the study website as belonging to a legitimate organization such as a university.

Success in recruiting participants online depends on many factors, which are similar to those for getting responses in traditional mail and telephone surveys. Studies have investigated various strategies to maximize response rates in offline surveys [36]. It is clear that there is no single strategy that can guarantee good response rates in all situations, due to variations in study characteristics, target populations, type and amount of incentives, sponsorships, length of questionnaires, text used for recruitment, and follow-up strategies. Future studies on Internet recruitment should focus on investigating ways to convey trust online to Internet users and to find attractive incentive structures for Internet users.
